# Long‐Term Stoma‐Free Outcomes After Diverting Stoma Closure Following Radiation‐Based Neoadjuvant Treatment for Rectal Cancer

**DOI:** 10.1002/ags3.70279

**Published:** 2026-09-08

**Authors:** Hidetaka Ichikawa, Shimpei Matsui, Kentaro Sato, Tatsuki Noguchi, Takashi Sakamoto, Toshiki Mukai, Tomohiro Yamaguchi, Takashi Akiyoshi

**Affiliations:** ^1^ Department of Colorectal Surgery Cancer Institute Hospital, Japanese Foundation for Cancer Research Tokyo Japan

**Keywords:** neoadjuvant therapy, radiotherapy, rectal neoplasms, stoma closure, total neoadjuvant therapy

## Abstract

**Aim:**

Long‐term stoma‐free outcomes after diverting stoma closure in patients who underwent sphincter‐preserving rectal cancer surgery with double‐stapling technique (DST) reconstruction following radiation‐based neoadjuvant treatment remain unclear. We evaluated delayed re‐stoma‐free survival, causes of delayed re‐stoma creation, and final stoma status.

**Methods:**

This retrospective cohort study included patients who underwent sphincter‐preserving rectal cancer surgery with double‐stapling technique reconstruction and diverting stoma creation following radiation‐based neoadjuvant treatment between 2011 and 2024. The primary outcome was delayed re‐stoma‐free survival after initially successful stoma closure. Delayed re‐stoma was defined as re‐stoma creation more than 30 days after closure.

**Results:**

Among 296 patients, 168 received radiotherapy without additional preoperative systemic chemotherapy (non‐TNT), and 128 received total neoadjuvant therapy (TNT). Stoma closure was achieved in 287 patients (97.0%). Five required re‐stoma creation within 30 days, leaving 282 with initially successful closure. During a median post‐closure follow‐up of 63 months, delayed re‐stoma creation occurred in 25 patients (8.9%): structural anastomotic complications in 14, functional disorders in seven, tumor recurrence in three, and dysmotility‐related megacolon in one. Estimated delayed re‐stoma‐free survival was 93.3% at 3 years and 90.3% at 5 years, with no significant difference between the non‐TNT and TNT groups (log‐rank *p* = 0.176). At the last follow‐up, 258 patients (87.2%) were stoma‐free.

**Conclusion:**

Approximately 90% of patients were stoma‐free at the final follow‐up. Delayed re‐stoma creation was most commonly attributable to structural anastomotic complications. Long‐term stoma‐free status should be considered a clinically meaningful endpoint beyond stoma closure alone.

## Abstract 2

This study evaluated long‐term stoma‐free outcomes in 296 patients who underwent rectal resection with double‐stapling technique (DST) reconstruction and diverting stoma creation following neoadjuvant radiotherapy‐based treatment. Among 282 patients who achieved successful initial stoma closure, the 3‐ and 5‐year freedom from late re‐stoma rates were 93.3% and 90.3%, respectively, with no significant difference between the TNT and non‐TNT groups. Male sex was independently associated with late re‐stoma.

## 1 Introduction

A diverting stoma is widely used to mitigate the clinical consequences of rectal anastomotic leakage after sphincter‐preserving surgery for rectal cancer [1]. However, stoma reversal is not achieved in all patients, and successful closure does not necessarily represent the final endpoint [2, 3]. Some patients subsequently require re‐stoma creation because of delayed anastomotic complications, severe defecatory dysfunction, tumor recurrence, or other late events. Therefore, long‐term stoma‐free status after closure is a clinically meaningful outcome beyond the closure rate alone.

This issue is particularly relevant in the era of intensified neoadjuvant treatment. Total neoadjuvant therapy (TNT) has become an important strategy for locally advanced rectal cancer because it enables systemic chemotherapy to be delivered before surgery, has yielded higher pathological complete response rates in Phase III trials, and may facilitate organ preservation in selected patients [4, 5, 6, 7]. At the same time, late radiation‐related tissue changes, including fibrosis and impaired tissue healing, remain potential concerns and may contribute to both structural anastomotic deterioration and progressive bowel dysfunction [8, 9]. However, long‐term stoma‐free outcomes after sphincter‐preserving surgery among patients treated with contemporary radiation‐based neoadjuvant strategies remain insufficiently characterized.

Delayed loss of stoma‐free status may result from heterogeneous pathways. Structural anastomotic complications, including delayed leakage, pelvic abscess, fistula, chronic sinus, and stenosis, may necessitate renewed fecal diversion. Severe functional disorders may also result in re‐stoma creation even in the absence of an identifiable structural abnormality. In routine clinical practice, re‐stoma creation after initially successful closure represents a robust and clinically ascertainable event reflecting structural, functional, oncologic, or other deterioration severe enough to require fecal diversion. Evaluating delayed re‐stoma therefore provides a patient‐relevant measure of sustained bowel continuity after sphincter‐preserving treatment.

The aim of this study was to characterize long‐term stoma‐free outcomes after diverting stoma closure in patients who underwent sphincter‐preserving rectal cancer surgery with double‐stapling technique (DST) reconstruction following radiation‐based neoadjuvant treatment. We specifically evaluated delayed re‐stoma‐free survival, causes of delayed re‐stoma creation, and final stoma status. We compared delayed re‐stoma‐free survival between patients who received radiotherapy without additional preoperative systemic chemotherapy (non‐TNT group) and those who received TNT and performed additional exploratory analyses according to radiotherapy schedule.

## 2 Methods

### 2.1 Study Design and Patients

This retrospective cohort study was conducted at the Cancer Institute Hospital of the Japanese Foundation for Cancer Research, Tokyo, Japan. We reviewed consecutive patients with rectal cancer who underwent definitive surgery between January 2011 and December 2024.

Eligible patients underwent sphincter‐preserving rectal resection with DST reconstruction and diverting stoma creation following radiation‐based neoadjuvant treatment. Patients with Stage IV disease at the time of surgery, those who underwent additional major procedures for other diseases during primary rectal surgery, and those who underwent reconstructive procedures other than DST reconstruction were excluded. A total of 296 patients were included.

Treatment strategy was determined at a multidisciplinary team conference. Radiation‐based neoadjuvant treatment consisted of either long‐course chemoradiotherapy or short‐course radiotherapy. Patients were classified into the non‐total neoadjuvant therapy (non‐TNT) group when they received radiotherapy without additional preoperative systemic chemotherapy and into the TNT group when systemic chemotherapy was administered before or after radiotherapy and before definitive surgery. Accordingly, the non‐TNT group included 135 patients who received long‐course chemoradiotherapy and 33 who received short‐course radiotherapy alone, whereas the TNT group included 94 patients treated with long‐course chemoradiotherapy‐based TNT and 34 treated with short‐course radiotherapy‐based TNT.

This study was approved by the Institutional Review Board of the Cancer Institute Hospital, Japanese Foundation for Cancer Research (approval No. 2018‐GA‐1030). The requirement for written informed consent was waived because of the retrospective design. The study was conducted in accordance with the Declaration of Helsinki.

### 2.2 Stoma Closure and Follow‐Up

Before stoma closure, all patients underwent contrast enema and endoscopic examination to evaluate the rectal anastomosis. When more than 6 months had elapsed since definitive surgery, surveillance computed tomography (CT) was also performed before closure. The timing of closure was determined according to the imaging findings and the patient's general condition. Closure was generally undertaken when no evident leakage, fistula, sinus, severe stenosis, tumor recurrence, or other finding considered unsuitable for restoration of bowel continuity was identified.

Patients were followed according to the institutional surveillance program after definitive rectal surgery and stoma closure. Data regarding stoma closure, re‐stoma creation, the indication for re‐stoma creation, final stoma status, tumor recurrence, and survival were collected from medical records.

### 2.3 Outcomes and Definitions

The primary outcome was delayed re‐stoma‐free survival after initially successful stoma closure. Initially successful stoma closure was defined as closure without re‐stoma creation within 30 days. Re‐stoma creation within 30 days was analyzed separately because it was considered to represent a rectal anastomotic abnormality that had not been detected during pre‐closure assessment but became clinically apparent shortly after restoration of bowel continuity. Delayed re‐stoma was defined as re‐stoma creation more than 30 days after closure.

Causes of delayed re‐stoma were classified as structural anastomotic complications, functional disorders, tumor recurrence, or other causes. Structural anastomotic complications were defined as objectively confirmed anatomical abnormalities directly leading to fecal diversion, including delayed leakage, pelvic abscess, fistula, chronic sinus, or clinically significant stenosis. Cases with an objectively confirmed structural anastomotic abnormality were classified as structural anastomotic complications, even when functional symptoms were also present. Functional disorders were defined only when severe bowel dysfunction required fecal diversion in the absence of structural anastomotic abnormalities or tumor recurrence sufficient to explain re‐stoma creation.

Final stoma‐free status was defined as the absence of a stoma at the last documented follow‐up, including patients who underwent successful re‐closure after re‐stoma creation. Permanent stoma status was defined as the presence of a stoma at the last follow‐up, regardless of whether the stoma resulted from nonclosure, early re‐stoma creation, or delayed re‐stoma creation. Preoperative malnutrition was defined as a serum albumin level < 3.5 g/dL, and anemia was defined as a hemoglobin level < 12.0 g/dL.

### 2.4 Statistical Analysis

Continuous variables are presented as medians with interquartile ranges and were compared using the Mann–Whitney *U* test. Categorical variables are presented as numbers and percentages and were compared using the chi‐square test or Fisher's exact test, as appropriate.

The cumulative probability of stoma closure and delayed re‐stoma‐free survival were estimated using the Kaplan–Meier method and compared using the log‐rank test. For the analysis of stoma closure, time was calculated from definitive rectal surgery to stoma closure, and patients who did not undergo closure were censored at the date of death or last follow‐up. For the analysis of delayed re‐stoma‐free survival, time was calculated from stoma closure to delayed re‐stoma creation among patients with initially successful closure. Delayed re‐stoma creation was considered the event. Patients who remained free from delayed re‐stoma were censored at the date of death or last follow‐up after closure, and death without delayed re‐stoma was not counted as an event. Three‐ and 5‐year delayed re‐stoma‐free survival rates were calculated from the Kaplan–Meier estimates.

Exploratory treatment comparisons included non‐TNT versus TNT, long‐course non‐TNT versus long‐course TNT, and long‐course versus short‐course radiotherapy‐based TNT. Characteristics of patients with and without delayed re‐stoma creation were compared using the Mann–Whitney *U* test, chi‐square test, or Fisher's exact test, as appropriate. These analyses were considered exploratory because of the limited number of delayed re‐stoma events.

All statistical tests were two‐sided, and *p* values < 0.05 were considered statistically significant. Analyses were performed using JMP version 18 (SAS Institute Inc., Cary, NC, USA).

## 3 Results

### 3.1 Study Cohort

**FIGURE 1:**
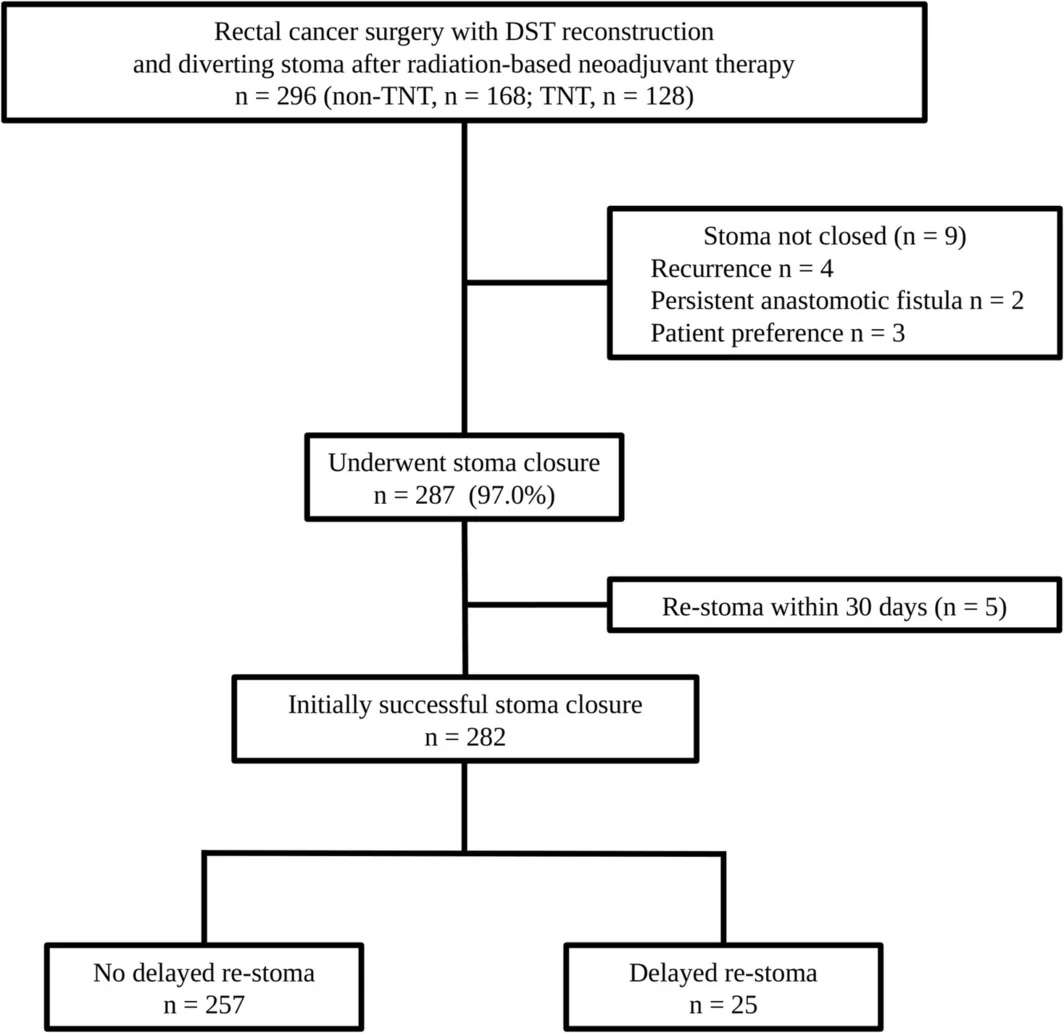
Patient flow according to long‐term stoma‐free status. Flow diagram of the study cohort, stoma closure status, re‐stoma creation within 30 days after closure, and delayed re‐stoma creation. DST, double‐stapling technique.

A total of 296 patients were included: 168 in the non‐TNT group and 128 in the TNT group (Figure 1). The non‐TNT group comprised 135 patients treated with long‐course chemoradiotherapy and 33 treated with short‐course radiotherapy alone, whereas the TNT group comprised 94 patients treated with long‐course chemoradiotherapy‐based TNT and 34 treated with short‐course radiotherapy‐based TNT. Patient, treatment, operative, and pathological characteristics are summarized in Table 1.

**TABLE 1:** Patient, treatment, operative, and pathological characteristics according to neoadjuvant treatment group.

Variable	Overall cohort *n* = 296	Non‐TNT *n* = 168	TNT *n* = 128	*p*
Baseline characteristics				
Age, years, median (IQR)	61 (52–68)	63 (54–70)	57 (50–66)	0.003
Male sex, *n* (%)	207 (69.9)	117 (69.6)	90 (70.3)	0.901
BMI, kg/m^2^, median (IQR)	23.0 (20.7–25.1)	23.0 (20.9–25.0)	23.2 (20.7–25.6)	0.253
ASA‐PS ≥ 3, *n* (%)	5 (1.7)	4 (2.4)	1 (0.8)	0.394
Diabetes mellitus, *n* (%)	40 (13.5)	27 (16.1)	13 (10.2)	0.140
Malnutrition, *n* (%)	22 (7.4)	13 (7.7)	9 (7.0)	0.818
Anemia, *n* (%)	86 (29.1)	46 (27.4)	40 (31.2)	0.468
Tumor and treatment characteristics before surgery				
Tumor distance from anal verge, cm, median (IQR)	5.0 (5.0–6.0)	5.0 (5.0–6.0)	5.0 (5.0–6.0)	0.503
Clinical stage, *n* (%)				< 0.001
I	1 (0.3)	1 (0.6)	0 (0.0)	
II	84 (28.4)	73 (43.5)	11 (8.6)	
III	211 (71.3)	94 (56.0)	117 (91.4)	
Treatment strategy, *n* (%)				
Long‐course CRT	135 (45.6)	135 (80.4)	—	
Short‐course RT alone	33 (11.1)	33 (19.6)	—	
Long‐course CRT‐based TNT	94 (31.8)	—	94 (73.4)	
Short‐course RT‐based TNT	34 (11.5)	—	34 (26.6)	
Radiotherapy schedule, *n* (%)				0.159
Long‐course	229 (77.4)	135 (80.4)	94 (73.4)	
Short‐course	67 (22.6)	33 (19.6)	34 (26.6)	
Operative characteristics				
Period of primary surgery, *n* (%)				< 0.001
2011–2017	163 (55.1)	119 (70.8)	44 (34.4)	
2018–2024	133 (44.9)	49 (29.2)	84 (65.6)	
Robotic approach, *n* (%)	57 (19.3)	16 (9.5)	41 (32.0)	< 0.001
Lateral pelvic lymph node dissection, *n* (%)	76 (25.8)	27 (16.1)	49 (38.6)	< 0.001
Operative time, min, median (IQR)	314 (252–390)	290 (242–357)	336 (281–437)	< 0.001
Blood loss, mL, median (IQR)	22 (10–70)	20 (10–50)	35 (10–100)	0.002
Anastomotic distance from anal verge, cm, median (IQR)	3.0 (3.0–4.0)	3.5 (3.0–4.0)	3.0 (3.0–4.0)	0.442
Pathological characteristics				
Pathological stage, *n* (%)				0.417
pCR/Stage 0	51 (17.2)	27 (16.1)	24 (18.8)	
I	79 (26.7)	48 (28.6)	31 (24.2)	
II	83 (28.0)	51 (30.4)	32 (25.0)	
III	83 (28.0)	42 (25.0)	41 (32.0)	

*Note:* Percentages were calculated using available data. Missing values: tumor distance from the anal verge, *n* = 1; lateral lymph node dissection, *n* = 1; anastomotic distance from the anal verge, *n* = 3. *p* values: Mann–Whitney *U*, chi‐square, or Fisher's exact test, as appropriate. Malnutrition was defined as serum albumin < 3.5 g/dL, and anemia was defined as hemoglobin < 12.0 g/dL.

Abbreviations: ASA‐PS, American Society of Anesthesiologists physical status; BMI, body mass index; CRT, chemoradiotherapy; IQR, interquartile range; RT, radiotherapy; TNT, total neoadjuvant therapy.

The median age was 61 years (interquartile range [IQR], 52–68 years), and 207 patients (69.9%) were male. Clinical Stage III disease was present in 211 patients (71.3%). Long‐course radiotherapy‐based treatment was administered to 229 patients (77.4%), and short‐course radiotherapy‐based treatment to 67 patients (22.6%). Robotic surgery was performed in 57 patients (19.3%), and lateral pelvic lymph node dissection in 76 patients (25.8%). The median anastomotic distance from the anal verge was 3.0 cm (IQR, 3.0–4.0 cm).

### 3.2 Stoma Closure and Early Re‐Stoma Creation

Stoma closure was achieved in 287 of 296 patients (97.0%; Table 2). Nine patients did not undergo stoma closure. Re‐stoma creation within 30 days after closure was required in five of the 287 patients who underwent closure (1.7%), leaving 282 patients with initially successful stoma closure. The cumulative probability of stoma closure did not differ significantly between the non‐TNT and TNT groups (log‐rank *p* = 0.395; Figure S1).

**TABLE 2:** Long‐term stoma‐related outcomes after diverting stoma creation and closure.

Outcome	Overall cohort
Initial stoma outcome after primary surgery	
Stoma closure achieved, *n* (%)	287/296 (97.0)
Stoma not closed, *n* (%)	9/296 (3.0)
Tumor recurrence	4
Persistent anastomotic fistula	2
Patient preference	3
Early outcome after stoma closure	
Re‐stoma creation within 30 days after closure, *n* (%)	5/287 (1.7)
Initially successful closure, *n* (%)	282/287 (98.3)
Delayed outcome after initially successful closure	
Delayed re‐stoma creation > 30 days after initially successful closure, *n* (%)	25/282 (8.9)
Structural anastomotic complications	14
Functional disorders without structural anastomotic abnormalities	7
Tumor recurrence	3
Dysmotility‐related megacolon	1
Final stoma status	
No delayed re‐stoma after initially successful closure, *n* (%)	257/282 (91.1)
Final stoma‐free status at last follow‐up, *n* (%)	258/296 (87.2)
Permanent stoma at last follow‐up, *n* (%)	38/296 (12.8)

*Note:* Initially successful closure was defined as absence of re‐stoma creation within 30 days after stoma closure. Final stoma‐free status included one patient who underwent re‐closure after delayed re‐stoma creation.

### 3.3 Delayed Re‐Stoma‐Free Survival

**FIGURE 2:**
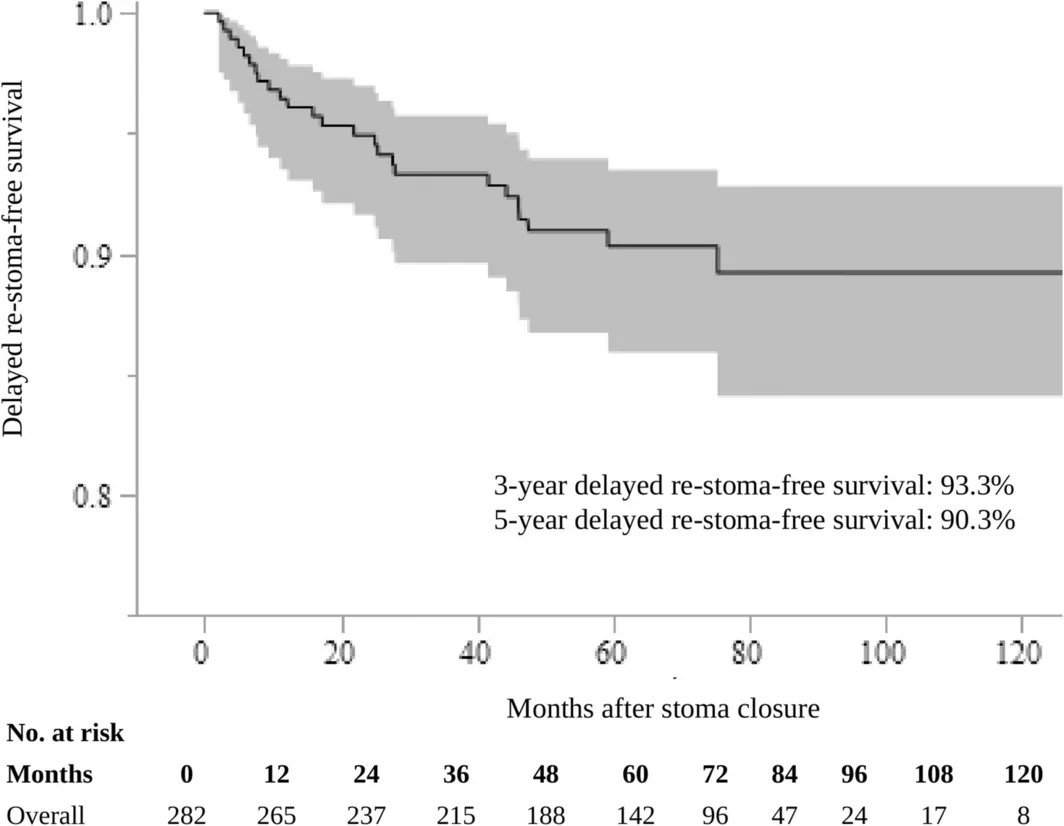
Overall delayed re‐stoma‐free survival after diverting stoma closure. Kaplan–Meier curve for delayed re‐stoma‐free survival among 282 patients with initially successful stoma closure. The shaded area represents the 95% confidence interval. The estimated delayed re‐stoma‐free survival rates were 93.3% at 3 years and 90.3% at 5 years.

During a median follow‐up of 63 months after stoma closure, delayed re‐stoma creation occurred in 25 of the 282 patients with initially successful closure (8.9%). The estimated delayed re‐stoma‐free survival rates were 93.3% at 3 years and 90.3% at 5 years (Figure 2).

Delayed re‐stoma‐free survival did not differ significantly between the non‐TNT and TNT groups (log‐rank *p* = 0.176; Figure S2). Additional exploratory analyses showed no significant difference between long‐course non‐TNT and long‐course TNT (log‐rank *p* = 0.427; Figure S3A) or between long‐course and short‐course radiotherapy‐based TNT (log‐rank *p* = 0.737; Figure S3B).

### 3.4 Exploratory Analysis of Factors Associated With Delayed Re‐Stoma Creation

Exploratory comparisons between patients with and without delayed re‐stoma creation are shown in Table 3. Male sex was more frequent among patients with delayed re‐stoma creation than among those without delayed re‐stoma creation (88.0% vs. 67.7%, *p* = 0.040). No statistically significant differences were observed in age, body mass index, diabetes mellitus, tumor distance from the anal verge, anastomotic distance from the anal verge, neoadjuvant treatment strategy, radiotherapy schedule, surgical approach, lateral pelvic lymph node dissection, anastomotic leakage after primary surgery, anastomotic stenosis or sinus before closure, adjuvant chemotherapy, or duration of follow‐up after stoma closure.

**TABLE 3:** Exploratory comparison of characteristics according to delayed re‐stoma creation after initially successful stoma closure.

Variable	No delayed re‐stoma *n* = 257	Delayed re‐stoma *n* = 25	*p*
Age, years, median (IQR)	61 (52–69)	58 (48–63)	0.094
Male sex, *n* (%)	174 (67.7)	22 (88.0)	0.040
BMI, kg/m^2^, median (IQR)	23.1 (20.5–25.0)	22.3 (21.1–24.3)	0.507
Diabetes mellitus, *n* (%)	37 (14.4)	2 (8.0)	0.548
Tumor distance from anal verge, cm, median (IQR)	5.0 (5.0–6.0)	5.0 (5.0–6.0)	0.640
Anastomotic distance from anal verge, cm, median (IQR)	3.5 (3.0–4.0)	3.0 (2.9–4.0)	0.257
Neoadjuvant treatment, *n* (%)			0.397
Non‐TNT	150 (58.4)	12 (48.0)	
TNT	107 (41.6)	13 (52.0)	
Radiotherapy schedule, *n* (%)			1.000
Long‐course RT‐based treatment	200 (77.8)	20 (80.0)	
Short‐course RT‐based treatment	57 (22.2)	5 (20.0)	
Surgical approach, *n* (%)			0.794
Laparoscopic	209 (81.3)	20 (80.0)	
Robotic	48 (18.7)	5 (20.0)	
Lateral pelvic lymph node dissection, *n* (%)	66 (25.7)	6 (24.0)	1.000
Anastomotic leakage after primary surgery, *n* (%)	8 (3.1)	2 (8.0)	0.219
Anastomotic stenosis or sinus before closure, *n* (%)	2 (0.8)	0 (0.0)	1.000
Interval from primary surgery to stoma closure, days, median (IQR)	156 (100–236)	193 (151–272)	0.050
Adjuvant chemotherapy, *n* (%)	106 (41.2)	15 (60.0)	0.090
Follow‐up after stoma closure, months, median (IQR)	63 (45–79)	63 (35–78)	0.767

*Note:* Continuous variables were compared using the Mann–Whitney *U* test. Categorical variables were compared using the chi‐square test or Fisher's exact test, as appropriate. Analyses were exploratory because of the limited number of delayed re‐stoma events.

Abbreviations: BMI, body mass index; IQR, interquartile range; RT, radiotherapy; TNT, total neoadjuvant therapy.

The median interval from primary surgery to stoma closure was 193 days among patients with delayed re‐stoma creation and 156 days among those without delayed re‐stoma creation (*p* = 0.050).

### 3.5 Causes and Timing of Delayed Re‐Stoma Creation

The causes and timing of delayed re‐stoma creation are summarized in Table 4. Structural anastomotic complications were the most common cause, accounting for 14 of the 25 delayed re‐stoma events. The median interval from stoma closure to re‐stoma creation in these patients was 11 months (IQR, 6–25 months).

**TABLE 4:** Cause‐specific details of delayed re‐stoma creation after stoma closure.

Cause of delayed re‐stoma creation	No. of patients	Time from stoma closure to re‐stoma, months, median (IQR)	Permanent stoma at last follow‐up, *n* (%)
Structural anastomotic complications	14	11 (6–25)	13 (92.9)
Functional disorders without structural anastomotic abnormalities	7	44 (20–47)	7 (100.0)
Tumor recurrence	3	11 (8–26)	3 (100.0)
Dysmotility‐related megacolon	1	46	1 (100.0)

*Note:* One patient with a structural anastomotic complication underwent re‐closure after delayed re‐stoma creation.

Abbreviation: IQR, interquartile range.

Functional disorders without structural anastomotic abnormalities accounted for seven events, with a median interval to re‐stoma creation of 44 months (IQR, 20–47 months). Three patients underwent delayed re‐stoma creation because of tumor recurrence, at a median of 11 months after closure (IQR, 8–26 months). One patient underwent re‐stoma creation 46 months after closure because of dysmotility‐related megacolon without anastomotic stenosis.

One patient with a structural anastomotic complication subsequently underwent successful re‐closure.

### 3.6 Final Stoma Status

At the last follow‐up, 258 of the 296 patients (87.2%) were stoma‐free, whereas 38 patients (12.8%) had a permanent stoma. The 258 patients with final stoma‐free status comprised 257 patients who had no delayed re‐stoma after initially successful closure and one patient who underwent successful re‐closure after delayed re‐stoma creation.

## 4 Discussion

This study evaluated long‐term stoma‐free outcomes after diverting stoma closure in patients who underwent sphincter‐preserving rectal cancer surgery with double‐stapling technique reconstruction following radiation‐based neoadjuvant treatment. Stoma closure was achieved in 287 of 296 patients (97.0%), and 282 patients had initially successful closure. Nevertheless, 25 patients (8.9%) subsequently required delayed re‐stoma creation. The estimated delayed re‐stoma‐free survival rates were 93.3% at 3 years and 90.3% at 5 years, and 258 patients (87.2%) were stoma‐free at the final follow‐up. These findings indicate that stoma closure alone does not fully represent long‐term treatment success and that maintenance of stoma‐free status should be assessed as a distinct, patient‐relevant outcome [2, 3].

The principal strength of this study is its focus on long‐term outcomes after initially successful closure rather than on the closure rate alone. Previous studies have mainly addressed nonclosure or morbidity associated with stoma reversal [2, 3], whereas data regarding loss of bowel continuity after apparently successful closure remain limited. By restricting the cohort to patients with double‐stapling technique reconstruction and a diverting stoma, we evaluated a relatively homogeneous population within a consistent reconstructive framework. Re‐stoma creation is also a robust clinical endpoint because it reflects deterioration severe enough to require renewed fecal diversion, irrespective of whether the underlying mechanism is structural, functional, oncologic, or otherwise.

Structural anastomotic complications were the most frequent cause of delayed re‐stoma creation, accounting for 14 of 25 events. Anastomotic leakage, chronic pelvic sepsis, fistula, sinus formation, stenosis, and other forms of anastomotic deterioration may remain clinically relevant long after restoration of bowel continuity [10, 11]. These late events are likely multifactorial and may reflect persistent pelvic sepsis, mechanical factors, impaired tissue healing, and progressive radiation‐related tissue changes, including fibrosis and microvascular injury [8]. Delayed anastomotic leakage may also represent either a previously occult leakage that becomes clinically apparent after stoma closure or newly developed anastomotic failure. Although delayed anastomotic abnormalities not requiring fecal diversion were also identified during follow‐up, the endpoint of the present analysis was restricted to clinically consequential events that ultimately required re‐stoma creation. Thus, the present findings should not be interpreted as representing the incidence of all delayed anastomotic abnormalities.

The substantial contribution of structural complications to permanent stoma formation emphasizes the importance of careful assessment before closure and continued vigilance after closure, even when no obvious abnormality is detected. Contrast enema and endoscopy were routinely performed before stoma closure, and when surveillance CT was performed, the anastomosis and surrounding pelvic structures were also carefully assessed for occult abnormalities. However, CT was not performed uniformly in all patients as a dedicated pre‐closure examination. Although cross‐sectional imaging may provide additional information in selected patients, the present study was not designed to evaluate its incremental diagnostic value or determine whether routine pre‐closure CT could prevent early re‐stoma creation.

Functional disorders accounted for seven delayed re‐stoma events and represented another important pathway to loss of stoma‐free status. Severe low anterior resection syndrome and other defecatory dysfunction can markedly impair quality of life and, in selected patients, ultimately lead to elective fecal diversion despite the absence of a demonstrable structural lesion [12, 13]. Progressive radiation‐related tissue changes may also contribute to long‐term deterioration of bowel function. In a long‐term follow‐up of a multicenter randomized trial, preoperative radiotherapy was associated with a higher prevalence of major low anterior resection syndrome 14 years after surgery [14]. Late radiation injury may therefore compromise long‐term stoma‐free status through both anastomotic damage and worsening bowel function. In this study, patients with an objectively confirmed structural anastomotic abnormality were classified as having a structural complication, even when functional symptoms were also present. Conversely, all seven patients classified as having functional disorders had no structural abnormality on endoscopy or imaging and required re‐stoma creation because of severe defecatory dysfunction. These cases show that anatomical continuity alone does not fully capture the functional success of sphincter‐preserving treatment. Future studies should include standardized patient‐reported functional measures alongside anastomotic and stoma‐related outcomes.

Randomized trials have established TNT as an important treatment strategy for locally advanced rectal cancer and have demonstrated higher pathological complete response rates and favorable oncologic outcomes [4, 5, 6]. TNT has also enabled organ preservation in selected patients [7]. In the present study, delayed re‐stoma‐free survival did not differ significantly between the non‐TNT and TNT groups. Similarly, no significant differences were observed according to radiotherapy schedule in the supplemental analyses. These findings provide no evidence that treatment intensification with TNT was associated with worse long‐term stoma‐free outcomes in this cohort. Nevertheless, treatment allocation was not randomized, treatment strategies evolved over time, and the subgroup analyses were limited by the number of events. Therefore, these findings should not be interpreted as proof of equivalence between treatment approaches.

Male sex was more frequent among patients with delayed re‐stoma creation than among those without delayed re‐stoma creation in the exploratory comparison. In contrast, TNT, radiotherapy schedule, lateral pelvic lymph node dissection, and anastomotic height were not significantly associated with the outcome. A narrower male pelvis and greater technical difficulty may contribute to anastomotic vulnerability or impaired pelvic drainage. However, the delayed re‐stoma group included heterogeneous structural, functional, and oncologic causes, and the number of events was limited. Accordingly, the observed association with male sex should be regarded as hypothesis‐generating rather than definitive.

This study has several limitations. First, it was a retrospective study conducted at a single high‐volume center, and treatment selection was influenced by changes in institutional practice during the long study period. Second, the number of delayed re‐stoma events was limited, restricting the precision of exploratory comparisons and subgroup analyses. Third, the study evaluated re‐stoma creation rather than all delayed structural or functional complications; clinically relevant events not requiring fecal diversion were therefore not reflected in the primary outcome. Fourth, patient‐reported bowel, urinary, and sexual function was not systematically available. Fifth, the decision to create a re‐stoma may have been affected by patient preference, functional tolerance, recurrence status, and physician judgment. Despite these limitations, this study provides clinically meaningful long‐term data after successful diverting stoma closure in a well‐defined cohort treated with contemporary radiation‐based neoadjuvant strategies.

## 5 Conclusion

Approximately 90% of the entire cohort were stoma‐free at the final follow‐up after radiation‐based neoadjuvant treatment and sphincter‐preserving rectal cancer surgery. Delayed re‐stoma creation occurred in 8.9% of patients after initially successful closure and was most commonly attributable to structural anastomotic complications. Delayed re‐stoma‐free survival did not differ significantly between the non‐TNT and TNT groups. Long‐term stoma‐free status should therefore be considered a clinically meaningful endpoint beyond stoma closure alone.

## Author Contributions

**Shimpei Matsui:** conceptualization, methodology, supervision, project administration, writing – review and editing, validation. **Kentaro Sato:** investigation, data curation, writing – review and editing. **Takashi Sakamoto:** data curation, investigation, writing – review and editing. **Toshiki Mukai:** data curation, investigation, writing – review and editing. **Tomohiro Yamaguchi:** methodology, conceptualization, supervision, writing – review and editing. **Tatsuki Noguchi:** data curation, investigation, writing – review and editing. **Hidetaka Ichikawa:** conceptualization, methodology, data curation, investigation, validation, formal analysis, visualization, writing – original draft, writing – review and editing. **Takashi Akiyoshi:** conceptualization, methodology, supervision, writing – review and editing.

## Funding

The authors have nothing to report.

## Ethics Statement

Approval of the research protocol by an Institutional Review Board: This study was approved by the Institutional Review Board of the Cancer Institute Hospital, Japanese Foundation for Cancer Research (approval No. 2018‐GA‐1030).

## Consent

The requirement for written informed consent was waived by the institutional review board because of the retrospective design.

## Conflicts of Interest

The authors declare no conflicts of interest.

## Supporting information


**Figure S1:** Cumulative probability of diverting stoma closure according to neoadjuvant treatment group. Kaplan–Meier curves comparing the cumulative probability of stoma closure from the time of definitive rectal surgery between the non‐TNT and TNT groups. No significant difference was observed between the groups (log‐rank *p* = 0.395). non‐TNT, radiotherapy without additional preoperative systemic chemotherapy; TNT, total neoadjuvant therapy.

**Figure S2:** Delayed re‐stoma‐free survival according to neoadjuvant treatment group. Kaplan–Meier curves comparing delayed re‐stoma‐free survival after initially successful stoma closure between the non‐TNT and TNT groups. No significant difference was observed between the groups (log‐rank *p* = 0.176). non‐TNT, radiotherapy without additional preoperative systemic chemotherapy; TNT, total neoadjuvant therapy.

**Figure S3:** Delayed re‐stoma‐free survival according to radiation‐based treatment strategy. Kaplan–Meier curves comparing delayed re‐stoma‐free survival after initially successful stoma closure. (A) Comparison between long‐course chemoradiotherapy without TNT and long‐course chemoradiotherapy‐based TNT (log‐rank *p* = 0.427). (B) Comparison between long‐course and short‐course radiotherapy‐based TNT (log‐rank *p* = 0.737). CRT, chemoradiotherapy; TNT, total neoadjuvant therapy.

**Table S1:** Outcomes according to neoadjuvant treatment and radiotherapy schedule.

**Table S2:** Case‐level details and classification of delayed re‐stoma creation.

## Data Availability

The data that support the findings of this study are available from the corresponding author upon reasonable request. The data are not publicly available because of institutional and privacy restrictions.
