# Supplementary material for: Long‐Term Stoma‐Free Outcomes After Diverting Stoma Closure Following Radiation‐Based Neoadjuvant Treatment for Rectal Cancer
Source: Ann Gastroenterol Surg. 2026 Sep 8:10.1002/ags3.70279. Online ahead of print. doi: 10.1002/ags3.70279 (PMC13551077; doi:10.1002/ags3.70279)
Supplement: Supplementary file 1 [file AGS3-9999-0-s002.docx]

| **Supplemental Table 2. Case-level details and classification of delayed re-stoma creation** | | | | | |
| --- | --- | --- | --- | --- | --- |
| **Case** | **Treatment** | **RT schedule** | **Interval from closure to re-stoma, months** | **Principal clinical indication** | **Basis for classification** |
| **Structural anastomotic complications (n = 14)** | | | | | |
| Case 1 | Long-course CRT | Long-course | 7.8 | Anastomotic leakage with fistula | *CT and endoscopy: perianastomotic extraluminal air with fistula formation* |
| Case 2 | Long-course CRT | Long-course | 7.5 | Rectourethral fistula | *CT and clinical findings: rectourethral fistula* |
| Case 3 | Long-course CRT | Long-course | 27.6 | Anastomotic leakage with fistula | *CT and endoscopy: perianastomotic extraluminal air with fistula formation* |
| Case 4 | Long-course CRT | Long-course | 9.4 | Anastomotic leakage | *CT: perianastomotic extraluminal air* |
| Case 5 | Long-course CRT | Long-course | 75.4 | Anastomotic leakage with fistula | *CT and endoscopy: perianastomotic extraluminal air with fistula formation* |
| Case 6 | Long-course CRT | Long-course | 27.9 | Anastomotic leakage | *CT: perianastomotic extraluminal air* |
| Case 7 | TNT | Short-course | 12.2 | Anastomotic leakage | *CT: perianastomotic extraluminal air* |
| Case 8 | TNT | Short-course | 2.8 | Anastomotic leakage | *CT and endoscopy: anastomotic dehiscence* |
| Case 9 | TNT | Short-course | 25.5 | Rectourethral fistula | *CT and clinical findings: rectourethral fistula* |
| Case 10 | TNT | Long-course | 2.1 | Anastomotic leakage | *CT: perianastomotic extraluminal air and fluid collection consistent with anastomotic leakage* |
| Case 11 | TNT | Long-course | 3.7 | Anastomotic leakage with fistula | *CT and endoscopy: perianastomotic extraluminal air with fistula formation* |
| Case 12 | TNT | Long-course | 4.9 | Rectovaginal fistula | *Endoscopy: rectovaginal fistula* |
| Case 13 | TNT | Long-course | 17.2 | Anastomotic leakage with fistula | *CT and endoscopy: perianastomotic extraluminal air with fistula formation* |
| Case 14 | Short-course RT alone | Short-course | 21.9 | Anastomotic leakage | *CT: perianastomotic extraluminal air and fluid collection consistent with anastomotic leakage* |
| **Functional disorders without structural anastomotic abnormalities (n = 7)** | | | | | |
| Case 15 | Long-course CRT | Long-course | 25.0 | Severe defecatory dysfunction | *No structural abnormality or recurrence on endoscopy and imaging* |
| Case 16 | Long-course CRT | Long-course | 15.9 | Severe defecatory dysfunction | *No structural abnormality or recurrence on endoscopy and imaging* |
| Case 17 | Long-course CRT | Long-course | 59.4 | Severe defecatory dysfunction | *No structural abnormality or recurrence on endoscopy and imaging* |
| Case 18 | TNT | Short-course | 44.4 | Severe defecatory dysfunction | *No structural abnormality or recurrence on endoscopy and imaging* |
| Case 19 | TNT | Long-course | 6.5 | Severe defecatory dysfunction | *No structural abnormality or recurrence on endoscopy and imaging* |
| Case 20 | TNT | Long-course | 47.6 | Severe defecatory dysfunction | *No structural abnormality or recurrence on endoscopy and imaging* |
| Case 21 | TNT | Long-course | 46.2 | Severe defecatory dysfunction | *No structural abnormality or recurrence on endoscopy and imaging* |
| **Tumor recurrence (n = 3)** | | | | | |
| Case 22 | Long-course CRT | Long-course | 41.7 | Tumor recurrence | *CT, MRI, and PET/CT: obstructing pelvic recurrence requiring transverse colostomy* |
| Case 23 | TNT | Long-course | 5.7 | Tumor recurrence | *Colonoscopy, CT, and MRI: anastomotic recurrence requiring abdominoperineal resection* |
| Case 24 | TNT | Long-course | 11.1 | Tumor recurrence | *CT, MRI, and PET/CT: pelvic recurrence requiring total pelvic exenteration* |
| **Other cause (n = 1)** | | | | | |
| Case 25 | Long-course CRT | Long-course | 46.3 | Dysmotility-related megacolon without anastomotic stricture | *CT and colonoscopy: megacolon without mechanical obstruction or anastomotic stricture* |
| Classification was based on the principal condition directly leading to fecal diversion. Structural anastomotic complications were defined as identifiable anatomical abnormalities, including anastomotic leakage, fistula, chronic sinus, or clinically significant stenosis. Functional disorders were defined as severe bowel dysfunction requiring fecal diversion in the absence of a structural anastomotic abnormality or tumor recurrence sufficient to explain re-stoma creation. Cases with an objectively confirmed structural anastomotic abnormality were classified as structural anastomotic complications, even when functional symptoms were also present. CRT, chemoradiotherapy; CT, computed tomography; MRI, magnetic resonance imaging; PET/CT, positron emission tomography/computed tomography; RT, radiotherapy; TNT, total neoadjuvant therapy. | | | | | |
