# Supplementary material for: Long‐Term Stoma‐Free Outcomes After Diverting Stoma Closure Following Radiation‐Based Neoadjuvant Treatment for Rectal Cancer
Source: Ann Gastroenterol Surg. 2026 Sep 8:10.1002/ags3.70279. Online ahead of print. doi: 10.1002/ags3.70279 (PMC13551077; doi:10.1002/ags3.70279)
Supplement: Supplementary file 2 [file AGS3-9999-0-s003.docx]

| **Supplemental Table 1. Outcomes according to neoadjuvant treatment and radiotherapy schedule** | | | | |
| --- | --- | --- | --- | --- |
| **Outcome** | **Long-course CRT** | **Short-course RT alone** | **Long-course CRT-based TNT** | **Short-course RT-based TNT** |
| Patients in the study cohort, n | 135 | 33 | 94 | 34 |
| Stoma closure achieved, n (%) | 131 (97.0) | 31 (93.9) | 91 (96.8) | 34 (100.0) |
| Re-stoma creation within 30 days after closure, n (%) | 0 (0.0) | 0 (0.0) | 2 (2.2) | 3 (8.8) |
| Initially successful closure, n | 131 | 31 | 89 | 31 |
| Delayed re-stoma after initially successful closure, n (%) | 11 (8.4) | 1 (3.2) | 9 (10.1) | 4 (12.9) |
| Median follow-up after closure, months | 68.0 | 62.0 | 54.0 | 56.0 |
| 3-year delayed re-stoma-free survival, % | 94.5 | 96.6 | 91.9 | 89.8 |
| 5-year delayed re-stoma-free survival, % | 91.6 | 96.6 | 88.2 | 85.3 |
| Structural anastomotic complications, n | 6 | 1 | 4 | 3 |
| Functional disorders without structural anastomotic abnormalities, n | 3 | 0 | 3 | 1 |
| Tumor recurrence, n | 1 | 0 | 2 | 0 |
| Dysmotility-related megacolon, n | 1 | 0 | 0 | 0 |
| **Delayed re-stoma-free survival was estimated after initially successful stoma closure. Log-rank p = 0.368 for long-course CRT versus short-course RT alone; p = 0.737 for long-course CRT-based TNT versus short-course RT-based TNT; and p = 0.848 for all long-course versus all short-course radiotherapy schedules. These subgroup analyses were exploratory because of the limited number of delayed re-stoma events.** | | | | |
| **Percentages for stoma closure were calculated using the study cohort in each group; percentages for re-stoma creation within 30 days were calculated using patients who achieved stoma closure; and percentages for delayed re-stoma were calculated using patients with initially successful closure. CRT, chemoradiotherapy; RT, radiotherapy; TNT, total neoadjuvant therapy.** | | | | |
